# Supplementary material for: Oxide interface-based polymorphic electronic devices for neuromorphic computing
Source: Nat Commun. 2026 Apr 9;17:3406. doi: 10.1038/s41467-026-71642-2 (PMC13069107; doi:10.1038/s41467-026-71642-2)
Supplement: Supplementary file 1 — Supplementary Information [file 41467_2026_71642_MOESM1_ESM.pdf]

Supplementary Information:

Oxide Interface-Based Polymorphic Electronic Devices for Neuromorphic Computing

Soumen Pradhan[1*], Kirill Miller[1], Fabian Hartmann[1*], Merit Spring[1], Judith Gabel[1], Berengar Leikart[1], Silke Kuhn[1], Martin Kamp[1], Victor Lopez-Richard[2], Michael Sing[1], Ralph Claessen[1], Sven Höfling[1]

[1]Julius-Maximilians-Universität Würzburg, Physikalisches Institut and Würzburg-Dresden Cluster of Excellence ctd.qmat, Am Hubland, Würzburg, 97074, Bavaria, Germany.

[2]Universidade Federal de São Carlos, Departamento de Física, Street, São Carlos, 13565-905 , SP, Brazil.

*Corresponding author(s). E-mail(s):

soumen.pradhan@uni-wuerzburg.de.

fabian.hartmann@uni-wuerzburg.de.

Supplementary Note 1:

The role of symmetry of bias voltage with respect to the middle of the nanowire device can be confirmed by means of a push-pull measurement. Here, the voltages V_D and V_S = - V_D are applied to the drain and source contacts, respectively. During the measurement, the voltage, V_D is swept to – V_S and V_S from - V_S to V_D. Supplementary Figure 2 shows the corresponding current-voltage characteristics. The push-pull method provokes an almost point symmetrical characteristic around zero bias voltage, and the memristive response is of a type-II character, as one expects in the case of symmetric carrier trapping processes [1]. For the push-pull measurement with grounded gates, the maximum and minimum currents are 2.6 μA and -2.3 μA, respectively. The small current asymmetry is most likely caused by a slightly asymmetrical

device layout, unavoidably induced by the different processing steps. The current-voltage characteristic for grounded gates indicates a small potential barrier in the channel (inset: Supplementary Figure 2). For the floating gate operation, we observe a high resistance plateau around zero bias voltage (inset: Supplementary Figure 2). For the down-sweep (i.e., from +5 V to – 5 V), the characteristic shows symmetrical threshold voltages with ΔV_{th}^{down} = 0.78 V. For the up-sweep, the measured voltage difference increases up to ΔV_{th}^{up} = 1.22 V. The type-II memristive response is discernible by the area enclosed during a bias voltage sweep. Therefore, the memory content in the device is highly sensitive on not only the amplitude of V_D but also the symmetry of bias between the drain and source contact.

Supplementary Note 2:

we have performed the endurance test on a single device in transistor, memristive and memcapacitive configurations consecutively. The results will define non-volatility and operational limit of each functionality including information about interference of one functionality into another one if there is any. However, at first, in transistor configuration, the gate voltage (V_G) was swept between -2 and 3 V for 1×10^3 cycles keeping the drain voltage (V_D) fixed at 0.2 V. The transfer characteristics are shown in Supplementary Fig. 4. The transistor remains functional showing switching between "on" and "off" states with positive and negative V_G for all the measurement cycles. However, a very small variation in drain current (I_D) is observed with sweep cycles. Also, a small left shift in threshold voltage (V_T) is observed for initial few cycles and then stabilize with cycles with the average value of -0.160±0.015 V.

Next, we have performed the cycle measurements in memristive configuration. The drain voltage (V_D) was swept between ±5 V with sweep cycle 2×10^3 keeping both the lateral gates floating as shown in Supplementary Fig.5a. The maximum and minimum currents are shifted for initial few cycles but thereafter a minor variation in current is observed throughout the cycles. Most importantly, we see a continuous increment of currents instead of collapsing the current or hysteresis area. Also, R_{on} and R_{off} are calculated from the linear fit of the current variation near zero bias for backward and forward sweeps. The R_{off} value remains fixed at around 10-15 GΩ, while R_{on} slowly decreases in the MΩ range as can be seen from the backward sweep cycles. However, the calculated R_{off}/R_{on} value displayed in Supplementary Fig. 5b shows that initially the resistance ratio increases a bit faster while an almost constant incremental trend is observed for the following cycles. The initial variation originates from the

transients of the device performance whereas an incremental charge accumulation on the floating gates with sweep cycles results in constant increment of resistance ratio. The same behavior is also observed when we extract the hysteresis area for the positive and negative bias regions as can be seen in Supplementary Fig.5c. Therefore, the endurance test on memristor configuration confirms that our device does not deteriorate up to the measurement cycles of 2×10^3, instead the on/off ratio and hysteresis area increase with cycles.

Finally, we have performed V_D sweep cycles of 100 for the memcapacitance measurements with an AC voltage of amplitude 20 mV and frequency 10 Hz as shown in Supplementary Fig.6a. We see complete overlap of the C-V curves for all the cycle measurements. We also extracted the capacitance values at zero bias from the forward and reverse sweep directions (C_{low} and C_{high}). Supplementary Fig.6b displays the C_{low} and C_{high} values from all the sweep cycles and clearly shows no variation of them with increasing the cycle number.

Therefore, we can confirm that all the functionalities are stable with sweeping cycles. Most importantly, cycling one mode does not degrade to another mode operation.

Supplementary Note 3:

In our devices, the q2-DEG, formed at the interface between crystalline LAO and STO is the core of all functionalities. Therefore, the variability and stability of the polymorphic functionalities depend on the stability of q2-DEG. Trier *et al.* reported that the crystalline LAO layers of 6 u.c. exhibits negligible time-dependent variation of sheet resistance, in contrast to thinner crystalline films (≤ 4 u.c.) or amorphous LAO grown on STO substrate [2]. Following that report, we also consistently grow 6 u.c. crystalline LAO films on STO to ensure stable q2-DEG formation in our study. These devices have been operated for several years. As an example, we are showing the I_D-V_D hysteresis curves in memristive configuration of the device after fabrication, 3 and 5 years after the fabrication as presented in Supplementary Fig.7. Though the current range has decreased over the years, it maintains the memory functionality even 5 years after the fabrication of the devices which confirms long-term stability of the devices. Most importantly, those devices are kept in open air, instead of storing in a high vacuum or controlled atmosphere.

Supplementary Note 4:

To understand the output inefficiency for higher V_D in the one transistor one memcapacitor (1T1MC) circuit, V_D is swept from 0 to 4 V keeping the transistor switched 'on' at a fixed gate voltage of 3 V, as represented in Supplementary Fig.8. The results reveal that the output voltage (V_O) increases linearly with V_D upto ≈2.5 V, beyond which the slope changes, and the reverse sweep exhibits hysteresis. It can be explained as a tuning of the local voltage efficiency. Since V_G=3 V, a polarity inversion is expected in the potential difference at V_D≈3 V. We note that all these capacitances change drastically with polarity.

Supplementary Note 5:

To demonstrate the operation of our 1T1MC architecture in RC system, we quantify the temporal memory as a function of pulse width. Here, we also extracted the characteristic memory kernels, decay time constants directly from our time-resolved output-voltage (V_O) measurements under different pulse-width stimuli. As shown in Fig. 2c in the main manuscript, V_O measured after a single input pulse of varying width exhibits (i) an abrupt drop immediately after pulse termination followed by (ii) a multi-timescale relaxation tail that constitutes the device's short-term memory. A zoomed view of the relaxation (Supplementary Fig.10a) clearly reveals two distinct decay regimes. The memory kernel increases with increasing the pulse width. To quantitatively extract the memory kernels, we fitted the relaxation to a double-exponential function

$$V_O = A_1 + A_2 \exp(-t/\tau 1) + A_3 \exp(-t/\tau 2) \ldots\ldots\ldots\ldots (1)$$

where $\tau 1$ and $\tau 2$ represent the fast and slow decay constants, respectively. The fits are illustrated in Supplementary Fig.10a. The extracted time constants versus pulse width are plotted in Supplementary Fig.10b. The fast component $\tau 1$ increases monotonically with pulse width, meaning that the short-term memory kernel becomes progressively longer. This is a desirable feature in physical reservoirs, where input amplitude–dependent fading memory broadens computational richness. The slow component, $\tau 2$, remains nearly constant for short pulses but exhibits a sharp increase for pulse width approaching 0.9 s, indicating the onset of a second, slow dynamical mode that enhances temporal separability between different input patterns.

Supplementary Note 6:

Here, we describe the requirements of nonlinearity and fading memory for hardware reservoir computing systems. Jang *et al.* in their review article nicely compared the reservoir output with and without non-linearity and fading memory [3]. They showed the output for two pulse schemes of "1100" and "1010" which have different temporal arrangements of the same high ("1") and low ("0") signals. It was observed that the outputs are inseparable without both the non-linearity and fading memory, with non-linearity but without fading memory and without non-linearity but with fading memory. In contrast, it shows separable output only with non-linearity and fading memory. However, we also performed the same measurements for two types of pulse schemes "1000" and "1100" with different temporal arrangements using our device in transistor mode in series with a commercial capacitor (10 µF) without non-linearity as shown in Supplementary Fig.11a,b. The output shows independence with arrangement of pulses. Therefore, the device configuration fails to demonstrate the reservoir computing system.

Supplementary Note 7:

A comparison of energy analysis is shown here between 1T1MC and 1T1M-based RC system.

(a) Energy analysis for 1T1MC reservoir (memcapacitor-based):

During reservoir computing operation, a voltage V_{MC} = 2.14 V develops across the memcapacitor under a gate pulse V_G = 3 V and V_D= 4 V. At positive V_G, the measured memcapacitance at the memcapacitor node ~155 pF. Therefore, the charge stored in the memcapacitor, Q = $C_{MC} \times V_{MC}$. and the energy drawn from the fixed drain supply can be calculated as:

$$E_{Source} = \int V_D\, i(t)dt = \int V_D dQ = V_D.Q = V_D.C_{MC}.V_{MC} = 4 \times 155 \times 10^{-12} \times 2.14 \text{ J} = 1.32 \text{ nJ} \quad ....(2)$$

On the other hand, energy drawn from the transistor gate driver (considering both the charging and discharging pulse) must include the standard capacitive charging+discharging term:

$$E_{Gate} = C_G.V_G^2 = 155 \times 10^{-12} \times 3^2 \text{ J} = 1.39 \text{ nJ} \quad ....(3)$$

Thus, the total energy drawn per pulse for 1T1MC configuration is

$$E_{pulse,\ total} = E_{Source} + E_{Gate} = (1.32 + 1.39) \text{ nJ} = 2.71 \text{ nJ} \quad ....(4)$$

(b) Energy analysis for 1T1M reservoir (memristor-based):

Now, let us evaluate the energy consumption for one transistor, one memristor (1T1M)-based reservoir computing system. For comparison, we consider the same supply voltage for a single

input pulse. As shown in Fig. 3b in the main manuscript, the output current (I_D) reaches ~2.53×10^{-8} A for a single 150 ms V_G pulse at V_D =4 V

Here, the energy drawn from the V_D supply is:

$$E_{Source} = \int_0^{\tau} V_D i(t)dt = V_D.I_D.\boldsymbol{\tau} = 4\times2.53\times10^{-8}\times0.15 \text{ J} = 15.18 \text{ nJ}\ldots\ldots(5)$$

The gate driven energy remains:

$$E_{Gate} = 1.39 \text{ nJ}$$

And the total energy drawn per pulse is

$$E_{pulse,\ total} = E_{Source} + E_{Gate} = (15.18 + 1.39)\ nJ = 16.57\ nJ$$

Therefore, the memcapacitor-based reservoir consumes approximately six times less energy per pulse than the memristor-based reservoir under identical bias conditions.

Energy and power analysis in 1T1MC-based RC system:

Using the 4-bit input scheme (four pulses per inference):

$$E_{inference} = 2.71\times4 \text{ nJ} = 10.84 \text{ nJ}.$$

The inference rate is

$$r = \frac{1}{4\times T} = 1.667 \text{ s}^{-1}$$

Average power: $P_{av} = E_{inference} \times r = 18.07$ nW

Supplementary Note 8:

As shown in Fig.2b in the main text, V_O depends on the input pulse width in a 1T1MC circuit. Therefore, to demonstrate the reservoir computing (RC) application of the circuit configuration, the width of the input pulses also plays a significant role. In the main text, the width of the pulses were kept fixed at 150 ms for the monochrome digit recognition task and all the 16 possible 4-bits pulse trains. Here, we measured V_O for all 16 states for pulse widths of 250 ms, 350 ms, 500 ms and 1 s, as shown in Supplementary Fig.12. The variation in reservoir states with different pulse widths reflects dynamic learning behavior, opening path for time series analysis.

Supplementary Note 9:

In human brain, continued or repeated stimulation results in long term storage of information. To mimic the same behavior, a presynaptic signal is applied with varying the width and number of the voltage pulses to the one transistor one memristor (1T1M) device structure as shown in Supplementary Fig.13a. Supplementary Figure 13b shows the input voltage (V_G) pulse of 3 V of different width and corresponding post synaptic current (PSC) for fixed V_D of 4 V. It is observed that there is an increase in PSC at the end of each pulse which corresponds to the increment of synaptic strength. Moreover, after returning the voltage pulse to 0 V, there is only a slow variation of PSC towards a relaxation value. Now, to better understand the results, change in PSC (ΔPSC) between beginning and end of a pulse is extracted and shown in Supplementary Fig.13c for different pulse width. With increase in pulse width, ΔPSC increases in a non-linear way which indicates the transition from short term memory (STM) to long term memory (LTM) in the device. Next, variation of input pulse number and corresponding PSC are shown in Supplementary Fig.13d. Similarly, PSC enhances with increase in pulse number. Here, the synaptic strength is extracted by evaluating the ratio of PSC of Nth pulse and 1st pulse (PSC_N/PSC_1) for each pulse train. Non-linear variation of PSC_N/PSC_1 with pulse number shown in Supplementary Fig.13e confirms the tuning possibility of synaptic strength like human brain. Also, before applying every pulse, PSC is reset to zero by applying a reset voltage of -2 V which is similar to resetting the memory of brain.

Supplementary Note 10:

The operation of a NOT gate is demonstrated using the device in transistor mode and a very high resistor (100 MΩ) compared to the "on" state resistance of the transistor in series as shown in Supplementary Fig.14a. The gate voltage (V_G) of the transistor is used as input signal and voltage between the resistor and transistor is considered as output signal (V_O). The drain voltage (V_D) of the transistor is kept fixed at 1 V during the logic operation. Now, to demonstrate the logic operation, V_G is swept from -1 V to 1 V. As depicted in Supplementary Fig.14b, when V_G is negative, V_O is high with a value of ~0.95 V at V_G = -1 V, then V_O decreases drastically when V_G approaches 0 V and at V_G = 1 V, V_O reaches ~0.05 V. Now, considering V_G of -1 V and 1 V as logic input "0" and "1", respectively, and V_O = 0.5 V as threshold voltage to distinguish between logic output "0" and "1", the operation of a NOT gate can be confirmed in a one transistor one resistor (1T1R) circuit.

Supplementary Note 11:

The logic computations and in-situ memory of logic output are demonstrated in two transistors one memristor (2T1M) device configuration utilizing one of the lateral gates from the two transistors as two input signals (Supplementary Fig.15a). To understand the origin of memory, gate voltages are swept between minimum and maximum values in forward and reverse directions [4]. Supplementary Figures 15b,c show the output current (I_{out}) for a gate voltage (V_G) sweep from -2 to 3 V and then 3 to -2 V of one transistor while V_G of the other transistor is kept fixed at -2 V for constant V_D of 4 V. It is observed that there is an anticlockwise hysteresis in $I_{out.}$ This is because the channel opens at around -1 V during forward sweep direction, and slowly current increases as we increase $V_{G,}$ and at V_G ~ 0 V, we see some current flow. In contrast, during reverse sweep cycle, a higher current is observed through the channel at V_G= 0 V which is due to the discharging of the floating gates in the whole circuit. Therefore, hysteresis in transfer characteristics is expected. Now, to confirm this, we have changed the V_G sweep rate and with increasing sweep rate there is a reduction in hysteresis since less time is spent above threshold voltage for faster sweep cycle to discharge charges to the channel from the floating gates.

Supplementary Note 12:

To quantify reset latency, we captured the current variation during reset process after each logic memory test for both the logic operations as shown at the end of each time measurement data in Figs.4c,d in the main manuscript. The decay of currents during reset processes are fitted with

the two exponential decay functions. This provides two-time constants $\tau 1$ and $\tau 2$, where $\tau 1$ directly corresponds to the reset time of logic output and $\tau 2$ indicates a short-term current memory of the device configuration. However, a chart of $\tau 1$ and $\tau 2$ values for both the logic operations are shown in Supplementary Figs.16a,b showing their statistical distribution. An average value of $\tau 1$ and $\tau 2$ are found ~0.05 and 1 s, respectively.

Supplementary Note 13:

The cycling endurance test defines the non-volatile behavior and operation limits including any drift in the logic operations in our 2T1M configuration. For logic OR and AND operations, we have arranged two transistors in parallel and series, respectively with one memristor in series. The endurance test of transistor and memristor functionalities already showed stable operations for all cycles without any drift. Moreover, in case of memristor, the resistance ratio between "off" and "on" states and hysteresis area slowly increase with increasing cycle number. This confirms the non-volatile behavior of the device functionalities. Therefore, it is obvious that the integration of transistors and memristor in our logic circuits will also be operational with endurance cycles. However, we have performed logic measurements with V_D sweep cycles to investigate the robustness of the logic output. For example, Supplementary Figs.17a,b show the output current with V_D sweep between 0 and 4 V with sweep cycles of 290 keeping both transistors switched "on" (logic input: "11") for logic OR and AND operations, respectively. The corresponding circuit diagrams are shown in the insets. The threshold current of 4 nA between logic output "0" and "1" is indicated by the dotted line in the figures. With increasing the sweep cycles, a minor variation of current is observed, but far above the threshold current range. Therefore, the robustness of the logic operations without any drift in our device configurations with non-volatile behavior is confirmed from the endurance cycle measurements.

Supplementary Note 14:

We conducted I_{out}-V_D full-cycle measurements between ±4 V using the circuit configuration shown in Supplementary Fig.18a, with input combinations-00, 10, 01, and 11. Here, the input "0" corresponds to V_G=-2 V and input "1" refers to V_G=3 V. As expected, hysteresis is observed in all cases except for the positive V_D sweep corresponding to IN-00, where the output current

remains very low (see Supplementary Fig.18b). Notably, during up-sweep at V_D =3 V, the I_{out} for IN-11 is significantly higher compared to IN-10 and 01, while during down sweep, all three inputs exhibit high I_{out} values. Therefore, by selecting an appropriate threshold current between these two sets of output levels, it is possible to construct an AND gate from an existing OR gate within the same circuit configuration. However, the AND gate operation is also shown from I_{out}-V_D full-cycle measurements between ±4 V using the circuit configuration shown in Supplementary Fig.19a. As we observe, there is negligible current flow in the positive side of V_D sweep cycle for IN-00, 10 and 01 (Supplementary Fig.19b). This is because the channel is depleted for these input combinations, resulting in "low" logic output. On the other hand, only for IN-11, the channel becomes open and a relatively high current is observed, making a "high" logic output.

Supplementary Note 15:

Our devices demonstrate the fundamental polymorphic behavior (transistor, memristor, memcapacitor) and their utility in neuromorphic circuits. A practical roadmap for LAO/STO-based lateral architecture can identify key constraints and the strategy for bridging from proof-of-concept to density, yield, and integration and here we outline the same.

Thermal budget considerations: The LAO/STO interface is sensitive to thermal cycling, particularly because high temperatures can drive oxygen vacancy migration, reconstruct the interface, or degrade the 2D electron gas. As such, we anticipate that any back-end processing (e.g., passivation, dielectric deposition, metallization) should be constrained to ≤ ~ 100–150 °C. To comply with this constraint, we propose using low-temperature deposition techniques such as plasma-enhanced atomic layer deposition (PE-ALD) to deposit dielectrics (e.g., HfO2, Al_2O_3, SiN_x) and encapsulation layers.

Interconnect and passivation strategy: For interconnects, viable materials include noble metals (e.g., Pt, Au) or Cu with diffusion barriers (TaN, TiN), deposited at low temperature to avoid damage to the oxide interface. Passivation and encapsulation are critical to ensure device stability and suppress environmental degradation; we favor ALD-grown Al_2O_3 as a conformal, low-diffusion, low-temperature passivation layer, possibly capped with a SiN_x for extra environmental protection.

Lithography and minimum feature size: The smallest features in our current devices are defined via electron beam lithography. To scale, conventional nano-lithography (e.g., E-beam or high resolution optical litho) can bring channel lengths to the 100-500 nm range, depending on the process. We estimate that a lateral pitch (gate-to-channel) of ~ 100-200 nm is feasible in the near term, allowing dense integration while preserving gate control and avoiding cross-talk.

Projected array density and yield: Assuming a conservative pitch of 250 nm and device footprint dominated by channel and gating area, we estimate a density on the order of $\sim 10^8$ devices/cm^2. Yield will critically depend on uniformity of the LAO/STO interface, lithographic variability, and contact reliability. To mitigate yield loss, we propose employing redundancy schemes (e.g., spare rows/columns, row/column error correction) and performing statistical process control combined with wafer-level electrical mapping to identify defect-prone regions early.

Integration with CMOS/system-level compatibility: We envision two main paths to integration with CMOS:

1. Heterogeneous integration: bonding a processed LAO/STO wafer onto a CMOS substrate (e.g., via flip-chip, hybrid bonding) to avoid disturbing the CMOS front-end.

2. Post-CMOS BEOL integration*:* if low-temperature dielectrics and metallization steps are carefully developed, it may be possible to process LAO/STO devices within the CMOS back-end-of-line (BEOL) flow. Critical to this will be controlling contamination (e.g., mobile ions), ensuring via formation compatibility, and maintaining planarity.

Finally, this roadmap demonstrates a credible path forward from lab-scale devices to small arrays, then to integration, and finally to reliability qualification.

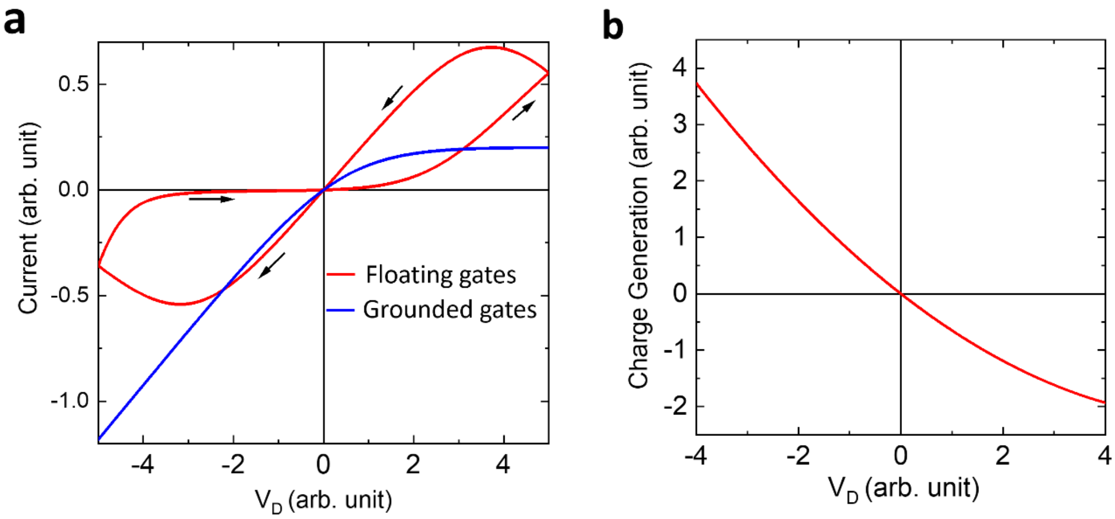

Supplementary Figure 1: Theoretical output characteristics of the device in memristive configuration*:* a, Simulated current-voltage characteristics for stable cycles under grounded gates (blue) and floating gate condition (red) according to the generation rate plotted in panel **b**.

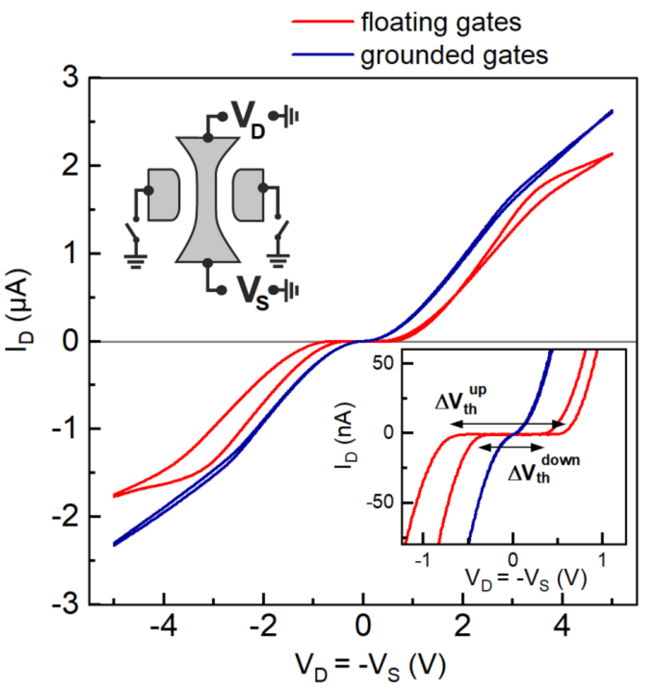

Supplementary Figure 2: Type-II memristive response: Current-voltage characteristics for the push-pull measurement with both the gates at floating and grounded conditions. The voltage V_D is applied to the drain contact and V_S (= - V_D) to the source contact. The current-voltage characteristic can be symmetrized and the memristor response can be tuned from type-I to type-II. The inset shows the resistance plateau region around zero bias for floating and grounded gate configurations highlighting different resistance plateau for up and down sweep directions at floating gate condition.

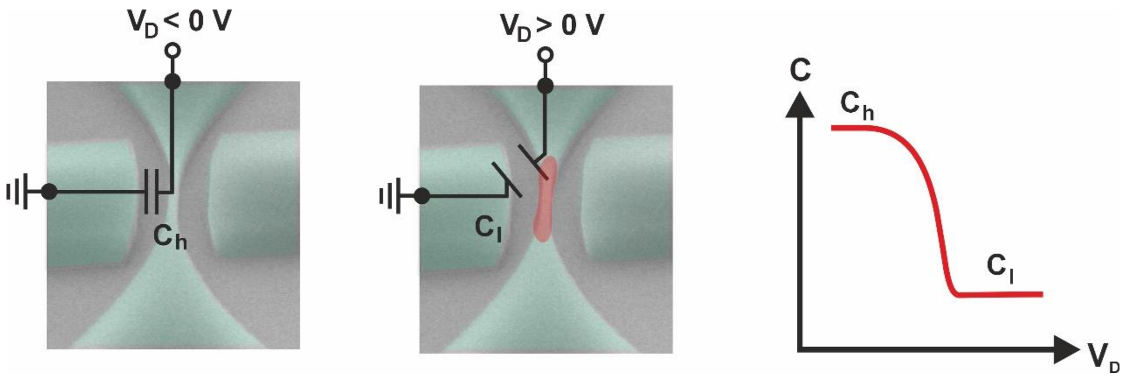

Supplementary Figure 3: Schematic representation of the two capacitance states: Capacitance representation under reverse and forward bias (V_D) condition in the memcapacitor configuration with schematic representation of transition of capacitance between high (C_h) and

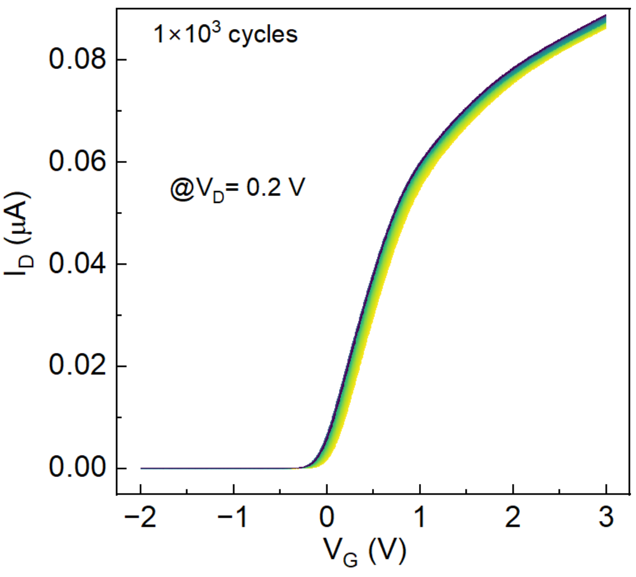

Supplementary Figure 4: Endurance test in transistor configuration: I_D-V_G curves in transistor configuration of the device for 1×10^3 cycles at V_D=0.2 V

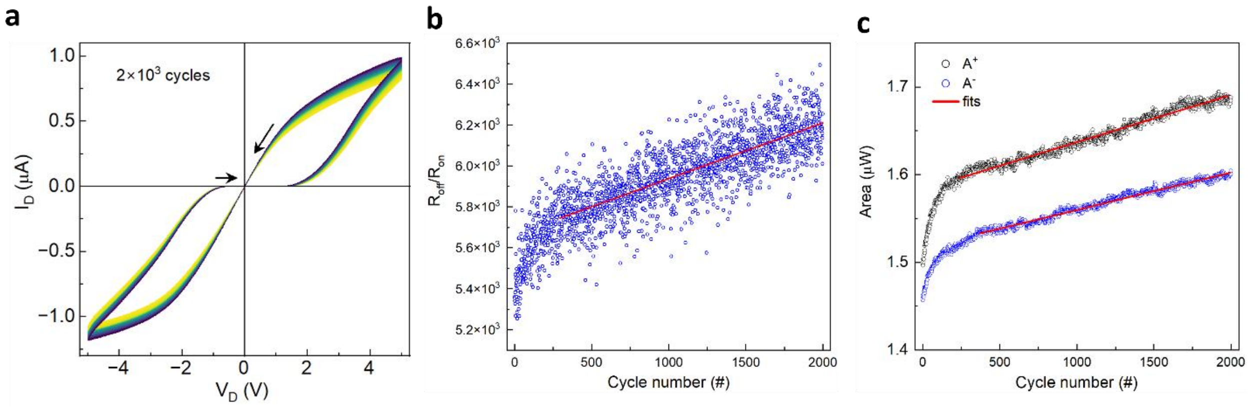

Supplementary Figure 5: Endurance test in memristor configuration: a, I_D-V_D hysteresis loops in memristive configuration of the device for sweep cycles of 2×10^3. **b,** Variation of R_{off}/R_{on} value with number of sweep cycles where R_{off} and R_{on} values were calculated from the linear fit of current variation near zero bias for forward and reverse sweeps, respectively. **c,** Variation of hysteresis area with cycle number for V_D>0 (A^+) and V_D<0 (A^-).

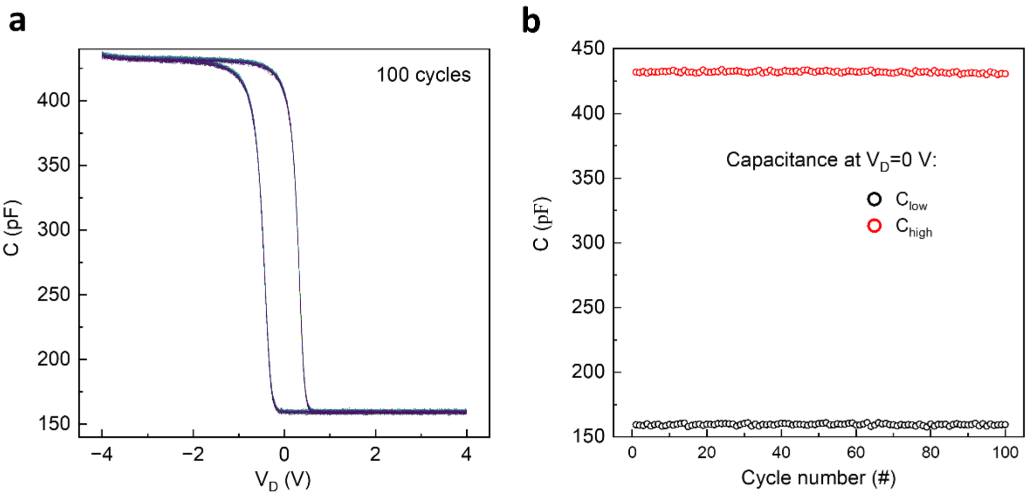

Supplementary Figure 6: Endurance test in memcapacitor configuration: a, C-V hysteresis curves with V_D sweep between ±4 V for 100 cycles keeping the auxiliary gate at floating condition measured at an AC voltage of 10 Hz. **b,** Variation of C_{low} and C_{high} with cycle number extracted from forward and reverse sweeps at zero bias.

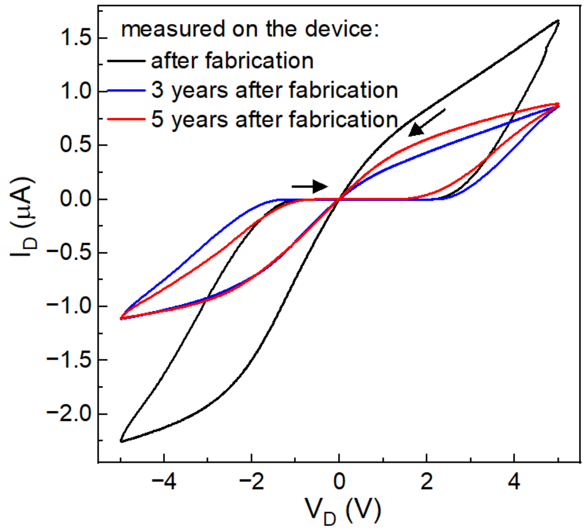

Supplementary Figure 7: Stability test in memristive configuration: Current-voltage (I_D-V_D) hysteresis curves measured in memristive configuration of the device after fabrication, 3 and 5 years after the fabrication.

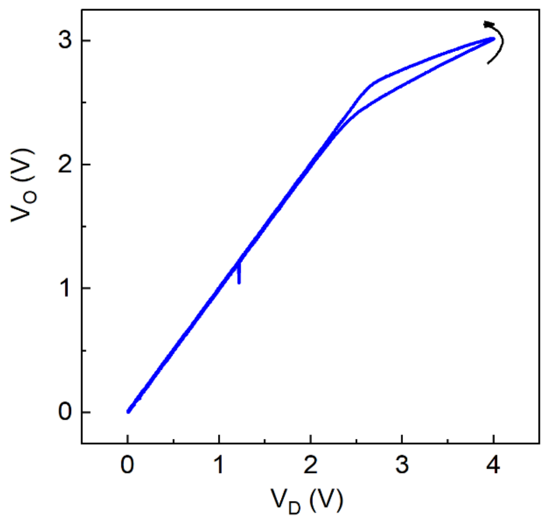

Supplementary Figure 8: Non-linearility and hysteresis in output voltage in 1T1MC circuit configuration: Hysteresis in V_O with V_D triangular sweep cycle between 0 and 4 V

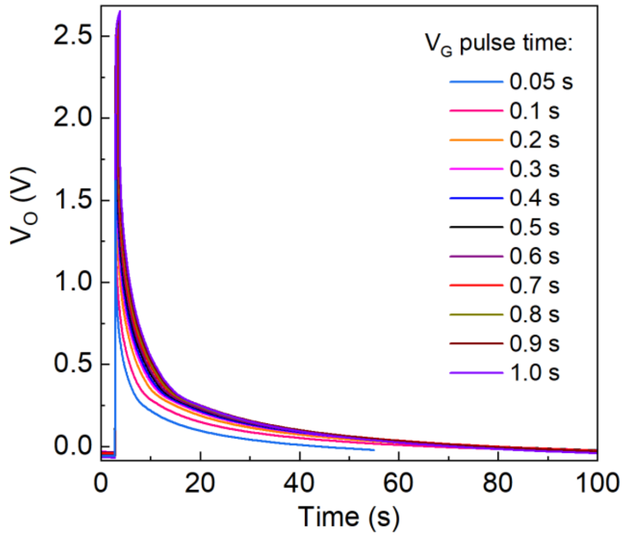

Supplementary Figure 9: Short-term-memory in 1T1MC circuit configuration: Output voltage (V_O) by applying single input pulse between -2 and 3 V of varying pulse width from 50 ms to 1 s at fixed V_D of 4 V. The decrement of V_O towards 0 V due to leakage of charges in the memcapacitor after switching off the transistor takes place in two steps: initially a very fast fall down followed by a comparatively slow process.

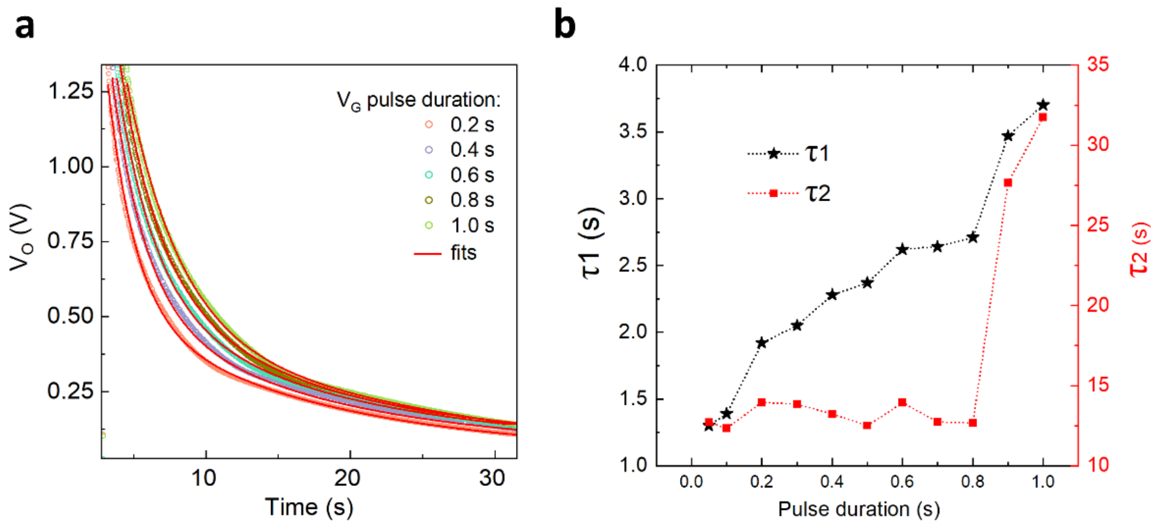

Supplementary Figure 10: Analysis of short-term-memory in 1T1MC circuit: a, A short window of V_O decay for a single input pulse of a few pulse widths at fixed V_D of 4 V with corresponding fits using two exponential decay functions, **b,** the variation of two decay constants with pulse duration.

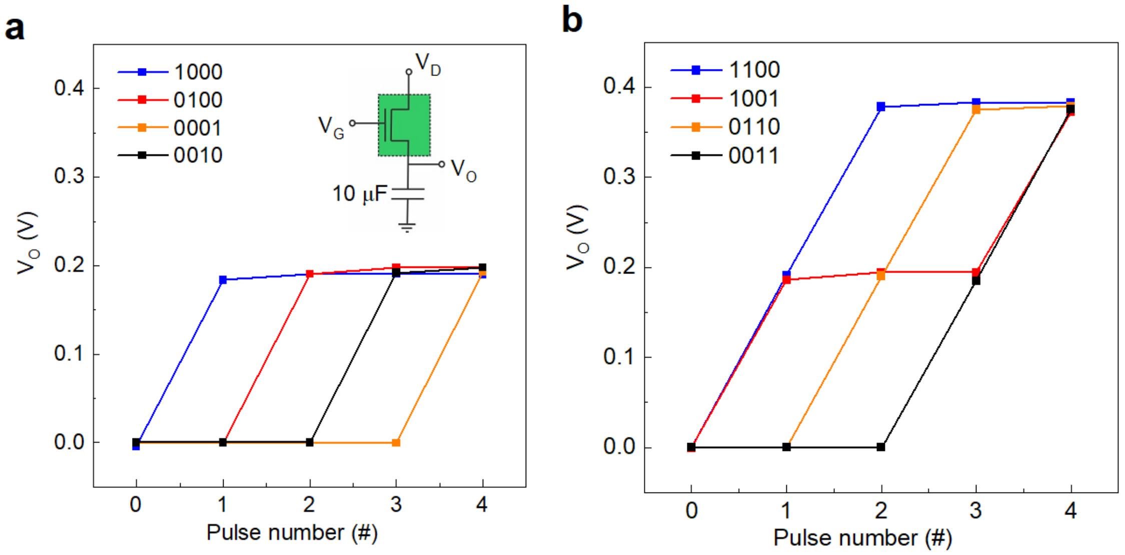

Supplementary Figure 11: Demonstration of Reservoir output without non-linearity: Output voltage (V_O) for 4-bit pulse train with pulse scheme of **a,** "1000" **b,** "1100" with different temporal arrangements of the same high ("1") and low ("0") signals applied to one transistor, one commercial capacitor (10 µF) circuit as shown in the inset of **a**.

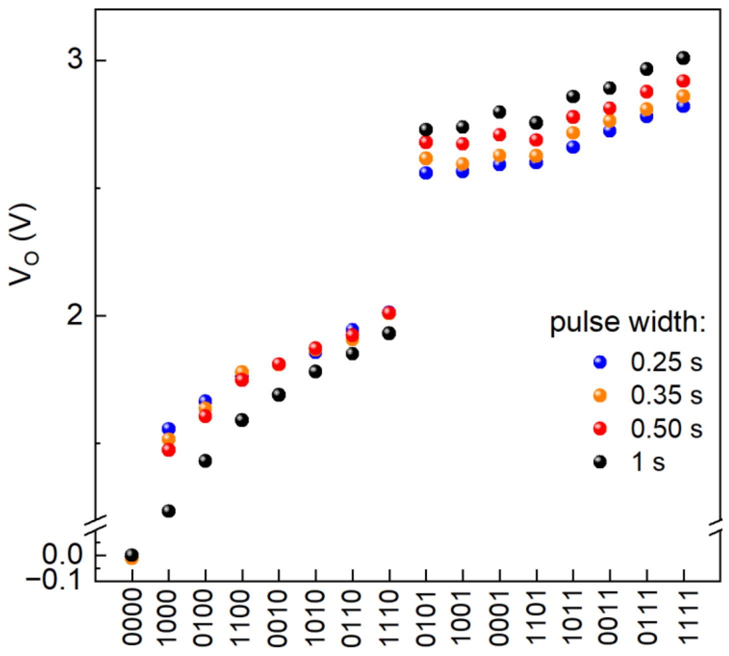

Supplementary Figure 12: Pulse width dependence of reservoir output: V_O at the end of all 16-types of 4-bit pulse trains applied to the input in 1T1MC circuit for different pulse width at fixed V_D of 4 V. Different pulse width corresponds to a different reservoir state, with almost all

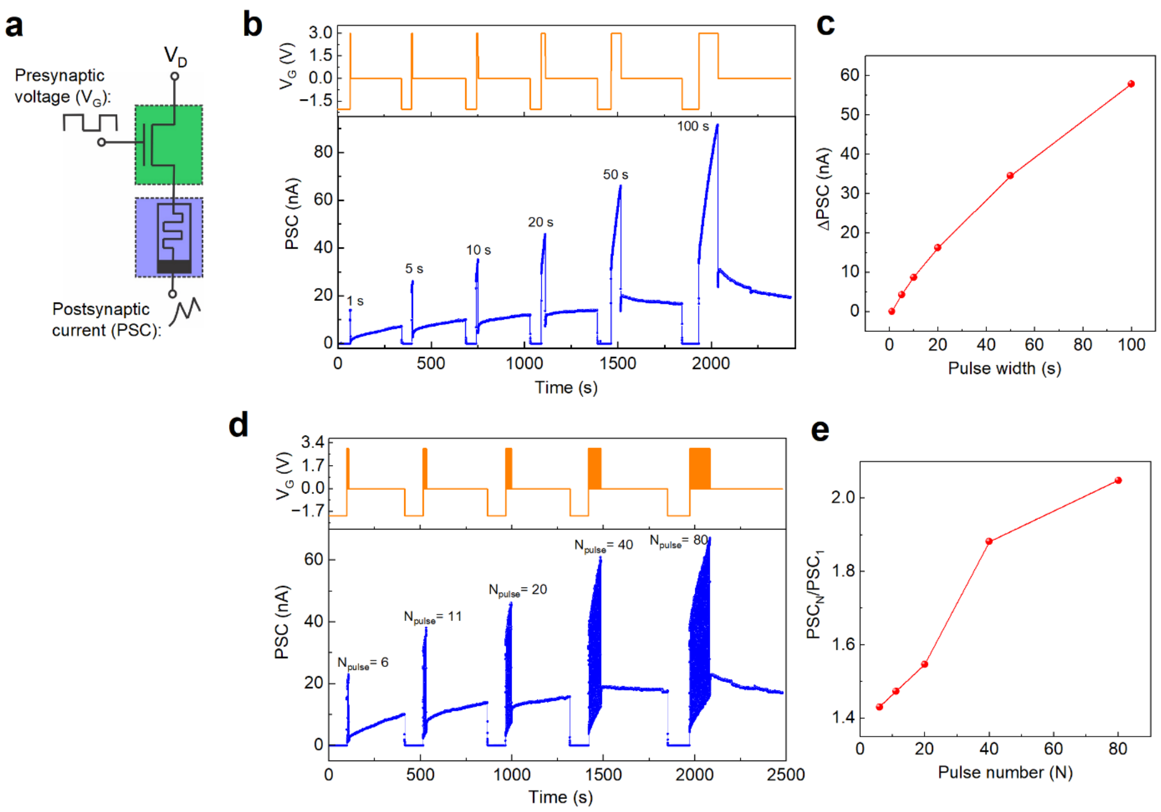

Supplementary Figure 13: Transition from STP to LTP in 1T1M device: a, schematic diagram of 1T1M device configuration with presynaptic voltage pulse to the gate of transistor and postsynaptic current (PSC) from the memristor, conversion from STP to LTP by increasing the input pulse **b,** width and **d,** number between -2 and 3 V at constant V_D of 4 V, **c,** plot of ΔPSC (difference between final and initial current during stimulation of a pulse) with pulse width and **e,** plot of PSC_N/PSC_1 with pulse number.

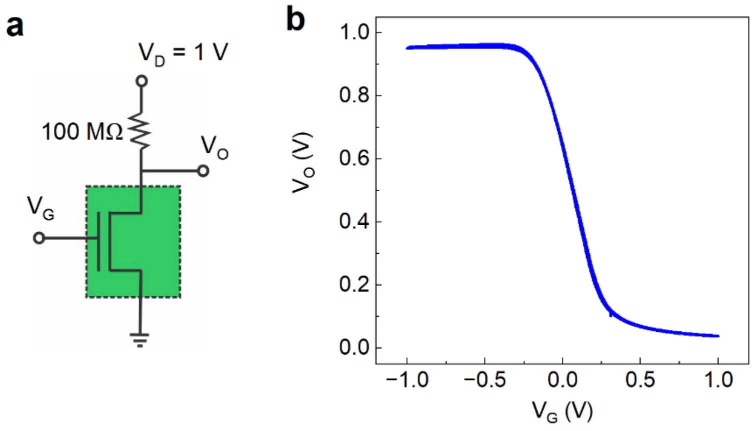

Supplementary Figure 14: NOT gate operation in 1T1R device: a, schematic illustration of 1T1R device configuration where one transistor is connected in series with one comparatively very high resistor; **b,** variation of output voltage (V_O) collected between the resistor and transistor as a function of gate voltage (V_G) at fixed V_D of 1 V. The data represents operation [illegible]

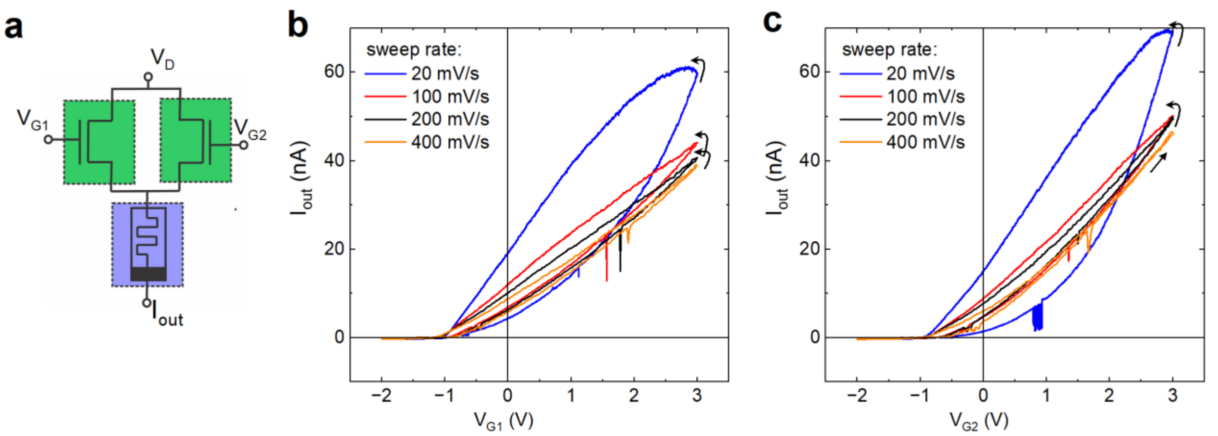

Supplementary Figure 15: Memory in transfer characteristics in 2T1M device: a, schematic diagram of 2T1M device configuration where two transistors, connected in parallel, are joined with one memristor in series, hysteresis in transfer characteristics curves for **b,** 1st transistor (left) keeping 2nd transistor (right) switched 'off' at V_{G2}= -2 V and **c,** 2nd transistor keeping 1st transistor switched 'off' at V_{G1}= -2 V for different sweep rate at fixed V_D of 4 V.

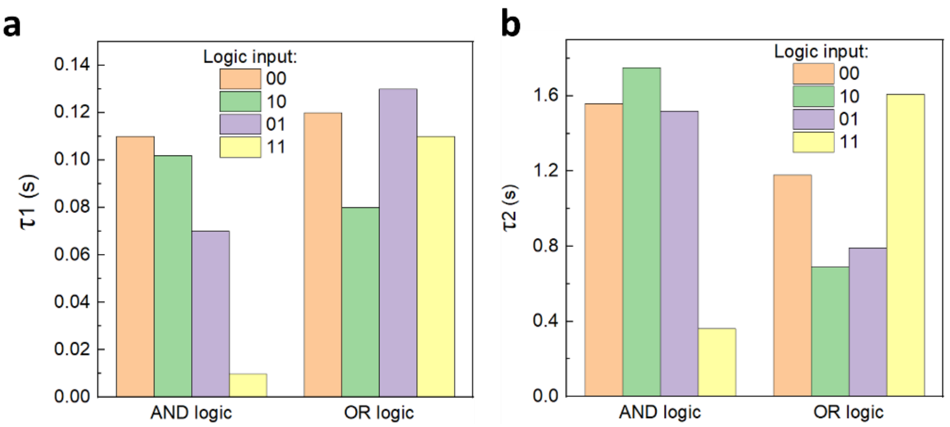

Supplementary Figure 16: **Reset analysis of logic output:** distribution of decay constant **a,** $\tau 1$ and **b,** $\tau 2$ for both the AND and OR logic operations extracted from the fits of reset processes shown in Fig. **4c** and **4d** with two exponential decay functions.

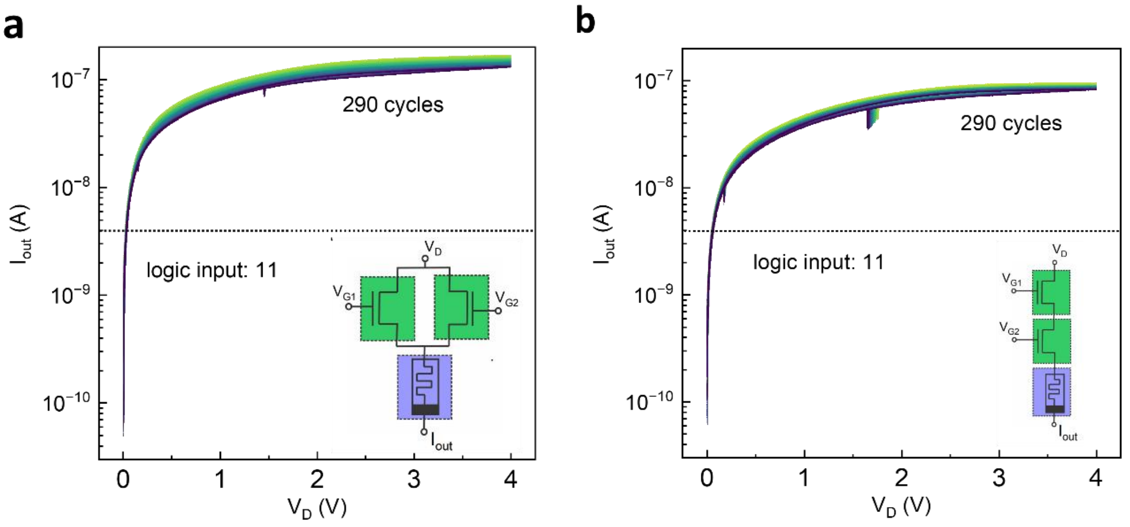

Supplementary Figure 17: Repeatability test of logic operations: Output current (I_{out}) with V_D sweep between 0 and 4 V with sweep cycles of 290 for logic input "11" (V_{G1}= V_{G2}=3 V) for **a,** OR and **b,** AND logic operations using 2T1M circuit diagram as shown in the insets. The dotted lines show the threshold current of 4 nA between logic output "0" and "1".

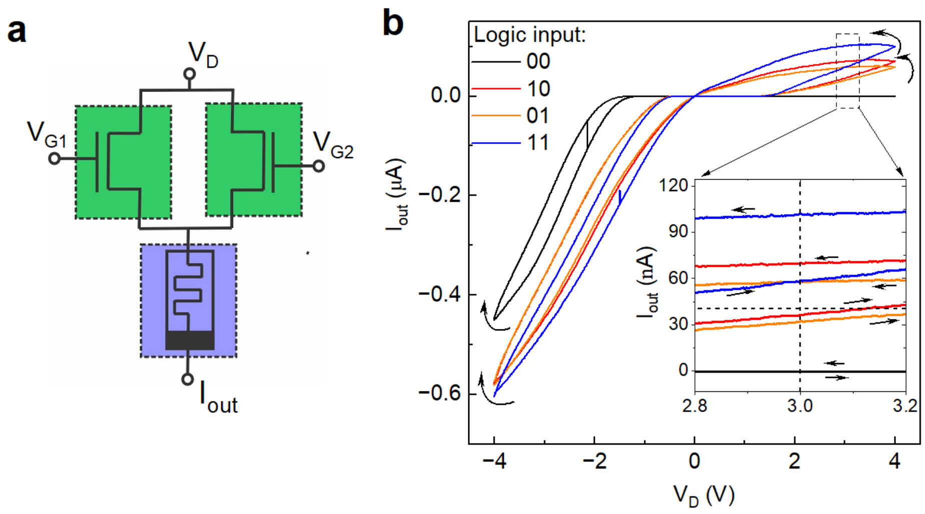

Supplementary Figure 18: **Logic OR operation in 2T1M device: a,** schematic diagram of 2T1M circuit diagram where two transistors in parallel configuration are connected with one memristor in series, and corresponding **b,** output current (I_{out}) with V_D sweep between ± 4 V for logic input "00" (V_{G1}= V_{G2}=-2 V), "10" (V_{G1}=3 V , V_{G2}=-2 V), "01" (V_{G1}=-2 V , V_{G2}=3 V) and "11" (V_{G1}= V_{G2}=3 V). In the positive side of V_D there is increment of current for logic inputs "10", "01" and "11" but not for "00" which confirms the logic OR operation. Inset shows the zoom-in plot around V_D = 3 V where forward sweep cycle for "10" and "01" can be distinguished from reverse sweep cycle for "10", "01" and "11" assuming a threshold current of 40 nA to be used for reconfigurable logic operation.

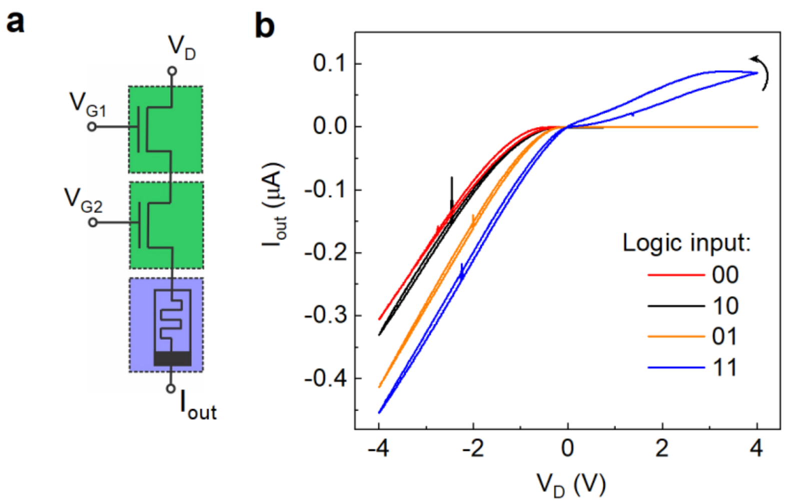

Supplementary Figure 19: Logic AND operation in 2T1M device: a, schematic diagram of 2T1M circuit diagram where two transistors and one memristor are connected in series, and corresponding **b,** output current (I_{out}) with V_d sweep between ± 4 V for logic input 00 (V_{G1}= V_{G2}=-2 V), 10 (V_{G1}=3 V , V_{G2}=-2 V), 01 (V_{G1}=-2 V , V_{G2}=3 V) and 11 (V_{G1}= V_{G2}=3 V). In the positive side of V_D there is increment of current only for logic input 11 which confirms the logic

Supplementary References:

1. Silva, Rafael Schio Wengenroth, Fabian Hartmann, and Victor Lopez-Richard. "The ubiquitous memristive response in solids." *IEEE Transactions on Electron Devices* 69.9 (2022): 5351-5356.
2. Trier, Felix, et al. "Degradation of the interfacial conductivity in $LaAlO_3/SrTiO_3$ heterostructures during storage at controlled environments." Solid State Ionics 230 (2013): 12-15.
3. Jang, Yoon Ho, Joon-Kyu Han, and Cheol Seong Hwang. "A review of memristive reservoir computing for temporal data processing and sensing." InfoScience 1.1 (2024): e12013.
4. Hou, Xiang, et al. "A logic-memory transistor with the integration of visible information sensing-memory-processing." *Advanced Science* 7.21 (2020): 2002072.
